# A Current Approach to Halitosis and Oral Malodor- A Mini Review

**DOI:** 10.2174/1874210601812010322

**Published:** 2018-04-30

**Authors:** Damla Aksit Bicak

**Affiliations:** Department of Pediatric Dentistry, Faculty of Dentistry, Near East University, Nicosia / TRNC Mersin 10 - Turkey

**Keywords:** Halitophobia, Halitosis, Oral malodor, Oral diseases, Pseudohalitosis, Volatile sulfur compounds

## Abstract

**Background:**

Halitosis, in other words, oral malodor is an important multifactorial health problem affecting the psychological and social life of individuals and is the most common reason for referral to dentists after dental caries and periodontal diseases.

**Objective:**

The objective of this review was to present and discuss conventional and recently introduced information about the types, causes, detection and treatment methods of halitosis.

**Methods:**

An expanded literature review was conducted which targeted all articles published in peer-reviewed journals relating to the topic of halitosis. Only articles written in Turkish and English languages were considered. The review itself began with a search of relevant subject headings such as ‘halitosis, oral malodor, volatile sulfur compounds in PubMed/Medline, Scopus, Google Scholar and Tubitak Ulakbim databases. A hand search of references was also performed.

**Results:**

When search results are combined, the total number of relevant literature was found to be 4646 abstracts and 978 full-text articles. Abstracts, editorial letters were not included and about half of full-text articles were not related to dental practice. Among the remaining 124 full-text articles, duplicated articles and articles written other than Turkish and English languages were removed and 54 full-text articles were used for this review.

**Discussion:**

According to the reviewed articles, both conventional and new methods were introduced in the management of halitosis. However, conventional methods seem to be more effective and widely used in the diagnosis and treatment of halitosis.

**Conclusion:**

As being first line professionals, dentists must analyze and treat oral problems which may be responsible for the patient's malodor, and should inform the patient about halitosis causes and oral hygiene procedures (tooth flossing, tongue cleaning, appropriate mouthwash and toothpaste selection and use) and if the problem persists, they should consult to a medical specialist.

## 1 INTRODUCTION

Halitosis is a general term describing bad or unpleasant smells from the oral cavity or outside the oral cavity. Halitosis came to fruition when the words “halitus” (breath) in Latin and “osis” (pathological process) in Greek came together [1]. Fetor ex-ore, fetor oris, oral malodor and bad breath all refer to unpleasant odors in the air of the expiration [2]. Oral malodor is a general term for bad odors that are emitted from the mouth, but it does not give information about the source and reason for the odor. The term ‘Oral malodor’ involves all of the oral odors such as ozostomia, stomatodysodia, halitosis and fetor oris / ex-ore. However, for differentiation, it is necessary to diagnose and determine the source of the odor [3]. The objective of this review is to present and discuss conventional and recently introduced information about the types, causes, detection and treatment methods of halitosis.

## 2 LITERATURE REVIEW/ DISCUSSION

### 2.1 Epidemiology

Halitosis is the most common reason for referral to dentists after dental caries and periodontal diseases [4]. According to a study in the Netherlands, halitosis was found among the 100 most common causes of distress in humans [5]. The prevalence of halitosis has been reported up to 50% in the literature. There are different prevalence values ​​due to differences in evaluation methods [6].

### 2.2 The Formation of Halitosis

Halitosis-forming gases are largely Volatile Sulfur Compounds (VSCs). These gases are hydrogen sulfide, methyl mercaptan, and dimethyl sulfide. Not only VSCs play a role in halitosis formation, at the same time, volatile aromatic compounds such as indole, skatole, organic acids such as acetic acid, propionic acid, and amines such as cadaverine and putrescin are also effective in the formation of halitosis. VSCs production in the oral cavity and its emergence depend on many local factors. These factors are saliva, decreased Oxygen (O_2_) concentration in the oral cavity, bacterial reproduction and metabolism [7].

### 2.3 Classification of Halitosis

Halitosis is divided into 2 groups as delusional (pseudohalitosis, halitophobia) and genuine halitosis. Genuine halitosis is divided into 2 subgroups as physiologic and pathologic halitosis. Pathologic halitosis can be oral or extraoral. Extraoral halitosis may originate from the respiratory system or other systems.

#### 2.3.1 Pseudohalitosis and Halitophobia

In pseudohalitosis, the patient complains of halitosis but the patient's oral malodor is not felt by others and the halitosis diagnosis can not be made objectively. In this case, explanation of halitosis and instructions for oral hygiene (support and reinforcement of a patient’s own self-care for further improvement of their oral hygiene), explanation of examination data, further professional instruction, education, and reassurance can be treatment choices for pseudohalitosis. Halitophobia is a fear that the patient's breath will be regarded as bad smell by other people. In halitophobia, the patient is worried about having continuous oral malodor. The patient believes that the oral malodor continues in spite of the treatment [4, 8]. As same as in the pseudohalitosis, the explanation of halitosis and instructions for oral hygiene, and also a referral to a clinical psychologist, psychiatrist or other psychological specialists can be treatment choices of halitophobia [4]. In the study of Tsuruta *et al*. [9], a total of 1360 female students answered a self-administered questionnaire regarding pathologic subjective halitosis, olfactory reference syndrome, social anxiety, and preoccupation with odors of body parts such as mouth, body, armpits, and feet. At the end of the study, researchers stated that social anxiety may be a causal factor of pathologic subjective halitosis and olfactory reference syndrome.

#### 2.3.2 Physiological Halitosis

Physiological halitosis is the transient bad oral malodor associated with nocturnal hyposalivation after sleep in the morning. There is no systemic disease or pathological condition that can cause halitosis. It develops due to bacterial activity during the night while sleeping [4, 8]. In a study of 55 children aged 3-14, there was an association demonstrated between oral respiration and oral malodor [10]. Physiological halitosis can be removed by correcting oral hygiene [11]. Transient halitosis can occur due to exogenous reasons (drinking alcoholic beverages, smoking, eating some foods). Smoking causes an increase in VSCs concentration in the mouth, hyposalivation and periodontal diseases. Alcohol causes hyposalivation. Foods such as onions and garlic have a high sulfur content. Sulfur passes blood circulation through the intestinal tract and felt as an odor during exhalation from the lungs [12, 13]. Explanation of halitosis and instructions for oral hygiene can be treatment choices for physiologic halitosis [4].

#### 2.3.3 Pathologic Halitosis

##### 2.3.3.1 Halitosis Originated from Oral Cavity

In 85% of patients with odor, the problem was found to originate from the bacterial activity in the oral cavity [14]. Some bacterial species, mainly gram-negative anaerobes are mostly responsible from halitosis. Odor components cannot form in the absence of microorganisms. Environmental factors are very important in the reproduction and growing of bacterial species [4]. A reduction in the amount of oxygen in the saliva and plaque plays an important and complex role in oral malodor formation. When the salivary flow rate decreases, bacterial count, and halitosis in the oral cavity increases [15]. The main causes of bad breath are tongue biofilm, bad oral hygiene, food impactions, candidiasis, soft diet, using orthodontic appliances, gingival and periodontal diseases (gingivitis, periodontitis, acute necrotizing ulcerative gingivitis, pericoronitis) dental abscess, hyposalivation due to medications, Sjogren syndrome, cancer treatment, bone diseases (alveolitis, osteomyelitis, osteonegrosis) and malign diseases [4].

There is usually a relationship between the amount of bacterial load on the tongue and oral malodor [16]. In individuals with healthy periodontal tissues and good oral hygiene, the posterior dorsum region is the main cause of oral malodor. Tongue coating may compose of desquamative epithelial cells, leukocytes from periodontal pockets, blood metabolites, different food residues, and bacteria. The surface of the tongue consists of papillaries and fissures thus the morphology of the tongue is extremely irregular. The morphological papillary structure of the dorsum of the tongue especially the depth of papillae influences the presence of tongue biofilm. This structure provides an appropriate anaerobic environment for bacterial growth, preventing the cleaning effect of saliva in these areas [17, 18]. In the cases of both periodontal diseases or healthy periodontal structures, bacteria colonize the tongue dorsum and periodontal pockets and play a major role in the formation of VSCs [19-22]. Mechanical removal of tongue biofilm has a crucial role in the control of halitosis. Tongue brushing or tongue scraping have the potential to successfully reduce breath odor and tongue coating [23]. With self-cleaning of tongue coating, halitosis could be decreased substantially [24]. In addition, the periodontal pocket is an ideal environment for VSC production with respect to the bacterial load and sulfur source. Also, VSCs accelerate periodontal tissue destruction. This is the reason of complaints of patients with periodontal diseases who have oral malodor. Maintaining good oral hygiene, rinsing with an effective mouthwash can be beneficial for oral malodor which originates from periodontal diseases. If the patients still suffer from oral malodor after these procedures, regular periodontal treatment such as Scaling and Root Planing (SRP) and a chlorhexidine mouth rinse usage are recommended for the treatment of oral malodor caused by periodontal diseases [25]. Caygur *et al*. [26] included 60 patients who presented with a 4- to 6-mm probing pocket depth in their study. Subjects were randomly selected for Scaling and Root Planning (SRP) or SRP + Glycine Powder Air-Polishing (GPAP). The plaque index, gingival index, pocket depth, bleeding on probing, and clinical attachment level scores were recorded for both groups at the beginning of the treatment and after 1 month. VSCs were measured by a Halimeter (Interscan Corp., Chatsworth, CA, USA) before the treatment, immediately after treatment, and at 7, 14, and 30 days. Researchers have indicated that GPAP does not provide any additional benefit to SRP in the treatment of periodontal diseases and oral malodor. Also, another contributing factor that significantly persuades halitosis is periimplantitis [27]. Tözüm *et al*. [28] reported a 58 years old female patient complaining of halitosis because of periimplantitis. After appropriate treatment of periimplantitis, patient’s complaints were recovered. Nani *et al*. [29] investigated the relationship among salivary bacteria, oral levels of VSCs, and stress in healthy male students and showed the stressed group had increased oral levels of hydrogen sulfide and dimethyl sulfide, together with higher salivary *Solobacterium moorei* levels. Bin Mubayrik *et al*. [30] measured self-perception, knowledge, and awareness of halitosis among 392 female university students by using a questionnaire and they were found that the participants not to have adequate knowledge and care about halitosis. For this reason, the researchers recommended increasing the role of dentists in informing and educating patients about oral malodor. Explanation of halitosis and instructions for oral hygiene, oral prophylaxis, tongue cleaning plus interdental flossing on morning breath, mouthwash (including amine fluoride, stannous fluoride, chlorhexidine) and toothpaste usage (stannous-containing paste), professional cleaning and treatment for oral diseases, especially periodontal diseases are treatment choices [4]. Different agents have been tried recently in the treatment of halitosis. For example, Melaleuca alternifolia oil can reduce bacterial growth and VSCs production and could be used as an alternative to chlorhexidine [31]. In another study, it was demonstrated that champignon extract made improvement in halitosis and body odor. It is stated that the effectiveness of champignon extract increases with the dosage [32]. Probiotics have also been shown to be beneficial in reducing bacterial populations in halitosis and periodontitis [33]. Also, homeopathy, herbal medicine and aromatherapy are used as alternative treatments, or complementary to conventional medicine in the treatment of halitosis [34]. In recent years, green tea has been used as an alternative method for treating halitosis. There are numerous beneficial effects of green tea (Camellia Sinensis) on oral health. Researchers suggest that green tea helps to reduce the bacterial activity in the oral cavity thus can reduce the formation of oral diseases [35, 36]. It is clear that new methods were introduced in the management of halitosis. However, conventional methods seem to be more effective and widely used in the diagnosis and treatment of halitosis.

##### 2.3.3.2 Extraoral Halitosis

###### 2.3.3.2.1 Halitosis Originated from Respiratory System

The odor is caused by nose and sinuses, Foreign Bodies (FB), tonsils, pharynx, and lungs. Diseases of the respiratory system cause the expiration of gas that gives off bad odor from the oral cavity and nose. The odor expired from the mouth and nose should be well distinguished. The existence of any FB in the nose causes inflammation, secondary infection and bad smell. Tonsillitis is one of the reasons of oral malodor in healthy individuals and also in children with cleft lip and palate. In the presence of *Pseudomonas auruginosa* in respiratory system diseases, bronchitis, bronchiectasis and lung diseases, 2-aminoacetophenone is excreted and this cause halitosis in adults [4].

###### 2.3.3.2.2 Halitosis Originated from Gastrointestinal System

Gastrointestinal diseases such as gastroesophageal reflux, gastric carcinoma, esophageal diverticulum can be the cause of halitosis. *Enterococcus faeccalis* and *Helicobacter pylori* (*H. pylori*) can be found in the periodontal pockets in the oral cavity and cause halitosis [4]. Moskowitz *et al*. [37] investigated gastrointestinal system diseases and halitosis association in 132 patients. They demonstrated a high correlation between the presence and severity of gastroesophageal reflux disease and halitosis. However, they did not find a similar relationship with peptic ulcer, dyspepsia, and *H. pylori* infections. Hoshi *et al*. [38] investigated the relationship between *H. pylori* and halitosis and used a rapid urease test for this purpose. At the end of the study, *H. pylori* was found positive in 31 of 80 patients. Gas chromatography results showed higher concentrations of dimethyl sulfide and hydrogen sulfide in the *H. pylori*-positive group.

Katsinoles *et al*. [39] observed that after eradication of *H. pylori* with 3 antibiotic treatment, most of the patients had decreased halitosis in long-term follow-ups. In another study [40], organoleptic scoring and the BANA test were both used to evaluate halitosis among gastric *H. pylori* positive and negative children. According to organoleptic scoring and BANA test, there was no clear relationship found between the gastric presence of *H. pylori* and halitosis. Among children who had *H. pylori* in their dental biofilm and saliva, BANA test positive results were found higher than children who did not harbor *H. pylori* in their oral cavities. BANA test detects *Treponema denticola, Porphyromonas gingivalis*, and *Tannerella forsythia* present on the dorsum of the tongue. The researchers concluded the reason for this result might be an increase in the oral prevalence of the VSCs producing periodontopathic microorganisms in the oral cavity with *H. pylori* colonization. *H. pylori* infection may be important in the pathophysiological mechanism of halitosis and *H. pylori* eradication therapy may be useful in patients with halitosis [41]. Oral malodor can also be caused by FB in the gastrointestinal system. Dedania *et al*. [42] reported a 58-year-old patient who did not recover from oral medical and dental hygiene procedures, and who did not have an important history with a halitosis complaint. Esophagogastroduodenoscopy was performed, showing the presence of a metallic FB in the form of a black wire embedded in the duodenum. FB was defined as a silver metallic flexible wire similar to that of a barbecue grill cleaning brush. The odor was completely resolved within 3 weeks after the FB was removed.

###### 2.3.3.2.3 Halitosis Originated from Metabolic Diseases

Metabolic diseases that can cause halitosis include diabetes, kidney failure, liver failure, trimethylaminuria, hypernatremia, and cystinosis [4]. Khozeimeh *et al*. [43] compared the concentration of urea and uric acid in patients with halitosis and without halitosis and found that salivary urea and uric acid concentrations greater in halitosis group than the control group which may responsible for oral malodor.

###### 2.3.3.2.4 Halitosis Originated from Drugs

In cases of using chemotherapy drugs, acetaminophen, chloral hydrate, dimethyl sulfoxide, disulfiram, nitrate and nitrites, and phenothiazines, halitosis can be observed. For halitosis originated extraorally, explanation of halitosis and instructions for oral hygiene and referral to a physician or a medical specialist can be treatment choices [4].

### 2.4 Halitosis Determination and Measurement Techniques

#### 2.4.1 Direct Measurement Techniques

##### 2.4.1.1 Organoleptic Measurement

Organoleptic measurement is a simple and widely used method. In this technique, a plastic tube is placed in the mouth of the patient and the patient is told to slowly breathe into the tube. During this time, the examiner evaluates the smell from the other side of the tube. This method increases reliability when used in conjunction with other measurement methods. In the most commonly used scoring system, the odor is classified between 0 and 5. (0: Odor cannot be detected. 1: Questionable malodor, barely detectable, 2: Slight malodor exceeds the threshold of malodor recognition. 3: Malodor is definitely detected, 4: Strong malodor, 5: Very strong malodor) [1].

##### 2.4.1.2 Gas Chromatography

The quantitative analysis of VSCs causing the odor (dimethyl sulfide, methyl mercaptan, and hydrogen sulfide gases) are performed by this method [8]. With this method, even low concentration of gases can be measured separately and their quantities can be determined [44]. In this method, samples are analyzed by a detector and mass spectra of existing compounds are compared and determined by a computer-based database. An automated aspiration system in gas chromatography has been developed to remove the differences caused by sampling or exhaling techniques [45]. Although gas chromatography is an objective method; there are some disadvantages such as being expensive, having non-transportable size and requires specialist personnel to use it. High correlations were found between organoleptic measurement and gas chromatography in the studies performed [8]. Oral Chroma^TM^ is a portable gas chromatographic device. The device can be used without any software [46].

##### 2.4.1.3 Portable Sulfide Monitor

The sulfide monitor is a portable device that allows easy measurement of the VSCs found in the expiration air outside the laboratory environment. The device was developed over time and presented to the market under the name of 'Halimeter’ (Interscan Corp., Chatsworth, CA, USA). With this method, the measurement is made as follows: The patient keeps his mouth closed for 5 minutes. Then, the patient inserts a single-use tube connected to the sulfide monitor into his mouth while breathing from the nose. The electrochemical reaction that takes place in the compounds containing sulfur in the breath brings the electric current in proportion to the levels of the compounds [44, 47].

#### 2.4.2 Indirect Measurement Techniques

##### 2.4.2.1 BANA (Benzoyl-DL-arginine- α-Naphthylamide) Test

Many of the anaerobic bacteria in the dorsum of the tongue and subgingival plaques can produce VSCs and malodorous volatile fatty acids. Detection of these bacteria and fatty acids can be used to diagnose halitosis. Proteolytic gram-negative anaerobic bacteria and short-chain volatile fatty acids, colonized in the subgingival plaque and the dorsum of the tongue, turn into a colored compound in the presence of the reducing enzyme, BANA, the synthetic trypsin substrate, and can be detected [48]. It is especially important to identify 3 major bacteria such as *Treponema denticola*, *Porphyromonas gingivalis*, and *Tannerella forsythia*. When these proteolytic bacteria are treated with a synthetic trypsin substrate BANA, the arginine hydrolase enzyme which is a colored compound is released. Thus, the presence of bacteria is proved. This test can easily be done with a 5-10 minutes period (BANA Test, Ora Tec, Manassas, VA / USA) [49].

##### 2.4.2.2 Chemical Sensors

Special probes have been developed to measure the amount of VSCs in the periodontal pocket and tongue surface. In the presence of sulfide ions, an electrochemical voltage is generated that is proportional to the sulfide-sensing element concentrations. This voltage is indicated by the digital score after the electronic unit is measured [50]. The electronic nose is another device developed for measuring halitosis. In this case, the information received by means of chemical sensors is transferred to the computer environment. It has 6 sensors with different selectivity and sensitivity. This method has a positive correlation with other measurement methods and is still in the developmental stage [51].

##### 2.4.2.3 Quantifying Beta-Galactosidase Activity

Levels of the enzyme beta-galactosidase have been found to correlate with oral malodor. The first step in the formation of halitosis is the glycosylation of glycoproteins. The activity of β-galactosidase, the most important enzyme of deglycosylation, can be assessed by impregnating the chromogenic substrate onto chromatography paper. When saliva comes in contact with the paper disc, a color change is detected on the paper according to the amount of enzymatic activity [52].

##### 2.4.2.4 Salivary Incubation Test

In this method, the saliva is collected in a glass tube and incubated at 37 °C; for several hours in an anaerobic medium containing 80% nitrogen, 10% carbon dioxide and 10% hydrogen. Then the odor is evaluated by the researcher. Saliva incubation test is less affected by external factors such as scented food eaten, fragrant cosmetic use, cigarette consumption than by organoleptic measurements [52].

##### 2.4.2.5 Ammonia Monitoring

It is a method based on the detection of ammonia released by oral bacteria which cause halitosis. The device consists of a pump that draws the expiratory air into the ammonia gas detector and a disposable tube that is inserted into the patient's mouth. In this method, the patients rinse their mouths with urea and then blow into the tube and the amount of ammonia is read by the gas detectors. The ammonia concentration produced by the bacteria can be read directly from the scale [53].

##### 2.4.2.6 Ninhydrin Method

Ninhydrin method is based on the detection of low molecular weight amines and polyamines that cannot be detected using the sulfur monitor. With this method, isopropanol is mixed with the sample taken from the patient and centrifuged. It is then read according to its light permeability using a spectrometer. Ninhydrin calorimetric reaction is fast, easy to apply and inexpensive [52].

##### 2.4.2.7 Polymerase Chain Reaction (PCR)

Polymerase Chain Reaction (PCR) is rapid, sensitive and specific diagnostic technique which has become an important and popular detection system in recent years. With using PCR, quantitative analysis of the microorganisms causing VSCs from oral specimens such as saliva, tongue coating, and subgingival plaque can be performed. Qualitative analysis methods are unsuitable for the accurate evaluation of bacteria causing oral malodor [54].

## CONCLUSION

Halitosis, also known as “bad breath” or “oral malodor,” is a major health problem that affects many individuals in population. Dentists are first-line health professionals in case of bad breath and play an important role in diagnosis, treatment, and referral the patients to a physician or a medical specialist if needed. It has a great importance to determine where the problem originates from the patient who has been admitted to dental clinic with a complaint of oral malodor. Taking anamnesis about the systemic diseases of the patient and making careful intraoral examination have great importance. The odor may be caused by the patient's oral cavity or may be a reflection of a previously undiagnosed or newly formed health problem. Sometimes the problem can be caused by the coexistence of oral and extraoral problems. Dentists must analyze and treat oral problems that may responsible of the patient's malodor, and should inform the patient about halitosis causes and oral hygiene procedures (tooth flossing, tongue cleaning, appropriate mouthwash and toothpaste selection and use) and if the problem persists, they should consult to a medical specialist.
